# A Silent Virus, a Dangerous Clot: A Case Report of Cytomegalovirus (CMV)-Associated Splanchnic Vein Thrombosis in an Immunocompetent Adult

**DOI:** 10.7759/cureus.105268

**Published:** 2026-03-15

**Authors:** Abay A Gobezie, Mekdem Bisrat, Samrawit W Zinabu, Sneha Adidam, Huda Gamelseed, Mrinalini Deverapalli, Miriam B Michael

**Affiliations:** 1 Internal Medicine, Howard University Hospital, Washington, DC, USA; 2 Infectious Diseases, Howard University Hospital, Washington, DC, USA; 3 Internal Medicine, Howard University, Washington, DC, USA; 4 Internal Medicine, University of Maryland, Baltimore, USA

**Keywords:** clinical case report, : cytomegalovirus, immunocompetent host, portal vein thrombosis, splenic infarction, superior mesenteric vein thrombosis, venous thromboembolism

## Abstract

Cytomegalovirus (CMV) infection typically presents as an asymptomatic or mild illness in immunocompetent hosts. However, thrombotic complications, though rare, have been increasingly recognized. We report a case of acute superior mesenteric vein (SMV) and portal vein thrombosis with splenic infarction in a previously healthy young adult with primary CMV infection. A 28-year-old male with no significant past medical history presented with acute pain in the left upper quadrant (LUQ) of the abdomen, fever, and diarrhea. Imaging revealed portal vein and SMV thrombosis with hepatosplenomegaly and multiple splenic infarcts. Laboratory evaluation was notable for lymphocytic leukocytosis and elevated liver enzymes. The thrombophilia workup was negative. CMV serology showed positive IgM antibodies and a significantly elevated CMV polymerase chain reaction (PCR) of 1,222 IU/mL, confirming acute primary infection. The patient was treated with anticoagulation and intravenous ganciclovir, resulting in clinical improvement. This report highlights the importance of considering CMV infection in the differential diagnosis of unusual venous thrombosis in immunocompetent young adults. Prompt recognition and treatment are essential to prevent severe complications. Clinicians should maintain a high index of suspicion for CMV-associated thrombosis when evaluating cases of thrombosis in atypical locations.

## Introduction

Cytomegalovirus (CMV) is a ubiquitous herpesvirus that infects between 40 and 100% of adults worldwide, depending on geographic region and socioeconomic factors [1]. In immunocompetent individuals, primary CMV infection is typically asymptomatic or presents as a self-limited, mononucleosis-like syndrome characterized by fever, malaise, and lymphadenopathy [2]. However, CMV has been increasingly recognized as a potential trigger for thrombotic complications, even among immunocompetent hosts [3].

The association between CMV infection and venous thromboembolism (VTE) was first described in the 1970s, but the underlying mechanisms remain incompletely understood [4]. Proposed pathophysiologic mechanisms include direct endothelial damage by viral invasion, increased expression of procoagulant factors, platelet activation, and generation of antiphospholipid antibodies [5]. Splanchnic vein thrombosis (SVT), including portal vein and superior mesenteric vein (SMV) thrombosis, represents a rare but potentially life-threatening manifestation of CMV infection [6].

We present a case of concurrent portal vein and SMV thrombosis with multiple splenic infarcts in an otherwise healthy 28-year-old male with acute CMV infection, highlighting the importance of considering this diagnosis in young patients presenting with unusual thrombotic events.

## Case presentation

Patient information and clinical history

A 28-year-old Spanish-speaking male with a medical history of obesity presented to the emergency department with a two-day history of sharp, severe left upper quadrant (LUQ) abdominal pain. The pain was rated 9/10 in intensity, radiated to the left flank and back, and was relieved by lying still. Associated symptoms included subjective fever, chills, nausea, and loose watery stools. The patient also reported intermittent exertional dyspnea that coincided with the abdominal pain.

Three weeks before presentation, the patient had experienced flu-like symptoms, including cough and congestion, with lingering symptoms at the time of admission. A coronavirus 2019 (COVID-19) test performed during that illness had been negative. He denied recent travel, sick contacts, chest pain, palpitations, dizziness, joint swelling, vomiting, dysuria, hematuria, hematochezia, or rashes. The patient had no known medical conditions and was taking no medications or herbal supplements.

Physical examination

On arrival, the vital signs were notable for tachycardia (heart rate 140 beats per minute), fever (102.5 °F/39.2 °C), and tachypnea (respiratory rate 20 breaths per minute). Blood pressure was within normal limits. Oxygen saturation was normal on room air. On general inspection, the patient appeared uncomfortable and in moderate distress due to pain. He was alert and oriented to person, place, and time. The patient was observed to prefer lying still in bed and demonstrated guarding behavior when attempting to change positions.

Head, eyes, ears, nose, and throat examination was unremarkable with moist mucous membranes and no scleral icterus. The neck was supple with no lymphadenopathy or jugular venous distension. Cardiovascular examination revealed tachycardia with regular rhythm and no murmurs, rubs, or gallops appreciated. Lung auscultation demonstrated clear breath sounds bilaterally with no wheezes, rales, or rhonchi, though respirations were mildly labored.

Abdominal examination revealed marked tenderness in the left upper quadrant to both superficial and deep palpation, with voluntary guarding noted. There was no rebound tenderness, rigidity, or percussion tenderness. Bowel sounds were present and normal in all four quadrants. No masses were palpable, and no organomegaly was appreciated on bedside examination. The spleen tip was not palpable. There was no costovertebral angle tenderness on percussion.

Extremity examination showed no peripheral edema, cyanosis, or clubbing. Peripheral pulses were intact and symmetric. The skin examination revealed no rashes, petechiae, or ecchymoses. Musculoskeletal examination demonstrated no joint effusions or limitations in range of motion. Neurological examination was non-focal with normal strength and sensation in all extremities.

Laboratory and diagnostic findings

Initial laboratory evaluation revealed significant abnormalities: white blood cell count of 18.4 × 10⁹/L (normal: 4.0-11.0) with an absolute lymphocytosis of 10.65 × 10⁹/L; elevated liver transaminases: alanine aminotransferase (ALT) 95 IU/L (normal <40), aspartate aminotransferase (AST) 61 IU/L (normal <40), and alkaline phosphatase (ALP) 146 IU/L (normal 30-120); normal total bilirubin, and a blood urea nitrogen/creatinine level of 1.26 mg/dL. The infectious workup, including HIV testing, T-SPOT tuberculosis assay, and an acute hepatitis panel (hepatitis A, B, and C), was negative. Blood cultures, stool cultures, a Clostridioides difficile assay, and stool tests for ova and parasites were negative. Fecal leukocytes were positive, suggesting inflammatory diarrhea.

**Figure 1:**
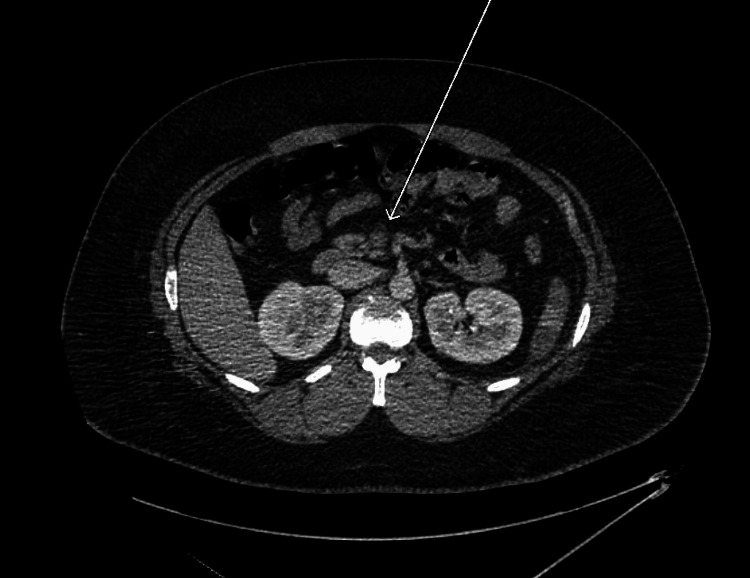
Axial CT image with intravenous contrast demonstrates a filling defect within the splenic vein (arrow), consistent with splenic vein thrombosis CT: computed tomography

**Figure 2:**
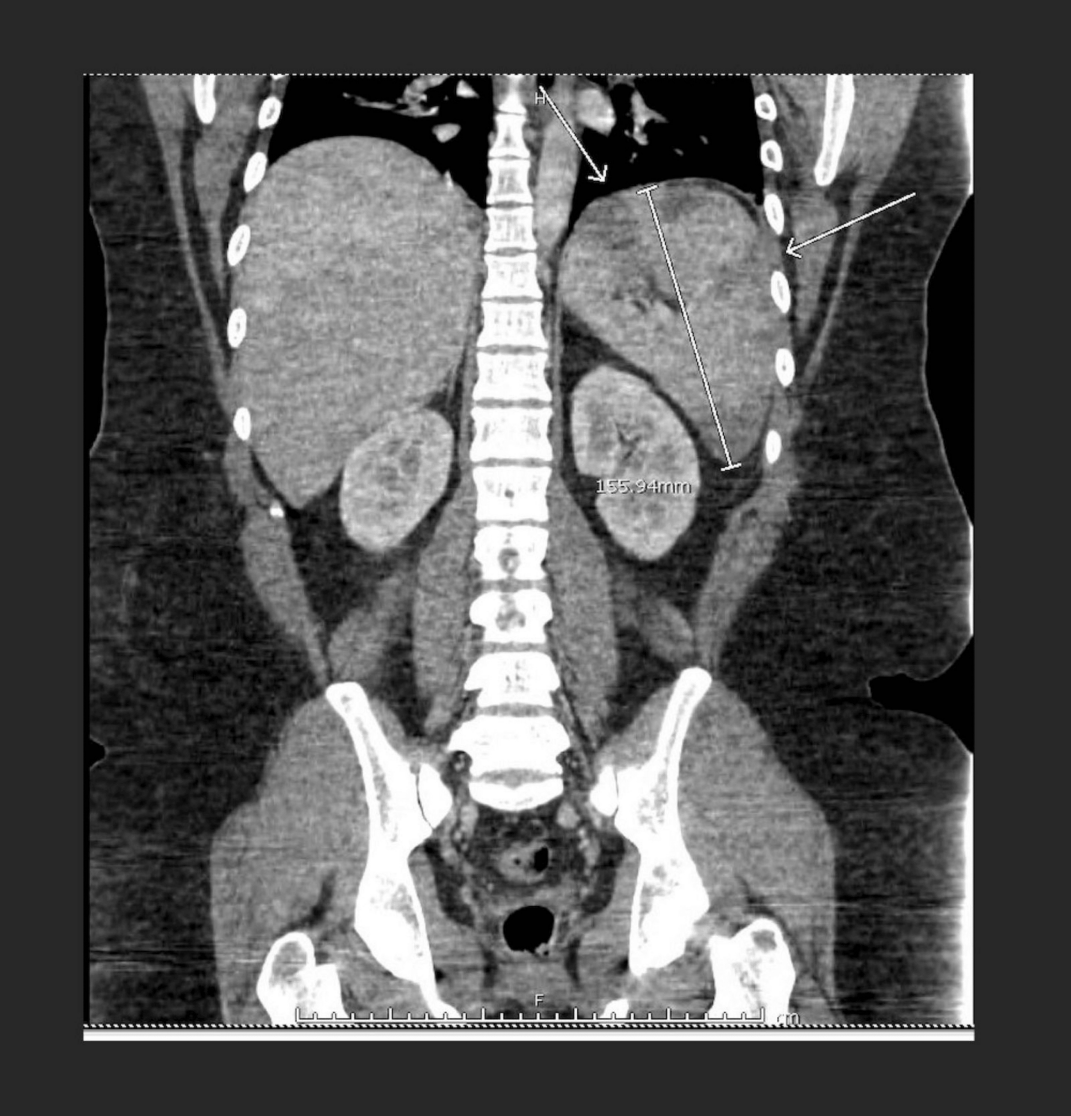
Coronal CT image demonstrates marked splenomegaly (white arrow) with multiple wedge-shaped hypodense areas consistent with splenic infarcts CT: computed tomography

Duplex ultrasound of the bilateral lower extremities was negative for deep vein thrombosis. CT angiography of the chest excluded pulmonary embolism. CT of the abdomen and pelvis with intravenous contrast revealed SMV and portal vein thrombosis, hepatomegaly, marked splenomegaly, and multiple wedge-shaped hypodense areas in the spleen consistent with splenic infarcts (Figures 1, 2).

Given the unusual presentation of splanchnic vein thrombosis in a young patient, comprehensive thrombophilia screening was performed and was negative for factor V Leiden mutation, prothrombin gene mutation, protein C deficiency, protein S deficiency, antithrombin deficiency, and antiphospholipid antibodies. Transthoracic echocardiography showed no evidence of infectious endocarditis or intracardiac thrombus. Viral serologic testing revealed positive CMV IgM antibodies with negative IgG antibodies, indicating acute primary infection. CMV polymerase chain reaction (PCR) quantification showed significantly elevated viremia at 1,222 IU/mL, confirming active CMV replication.

Treatment and clinical course

The patient was immediately initiated on therapeutic anticoagulation with intravenous unfractionated heparin for the management of portal vein and superior mesenteric vein thrombosis. Given the confirmed acute CMV infection with high viral load and evidence of end-organ involvement (hepatitis and splenic infarction), treatment with intravenous ganciclovir 5 mg/kg every 12 hours was initiated.

The patient demonstrated significant clinical improvement over the subsequent 48-72 hours with resolution of fever, decreased abdominal pain, and improvement in gastrointestinal symptoms. Laboratory parameters, including white blood cell count and liver enzymes, began to trend toward normal. After achieving therapeutic anticoagulation goals, the patient was transitioned from heparin to oral warfarin with a target international normalized ratio (INR) of 2-3. He was discharged home in stable condition on oral warfarin therapy and completed a 14-day course of intravenous ganciclovir administered via home health services. Long-term anticoagulation for a minimum of three to six months was recommended with close outpatient follow-up.

## Discussion

This report illustrates a rare but clinically significant complication of acute CMV infection in an immunocompetent host. While CMV is well-recognized as a cause of severe disease in immunocompromised patients, its association with thrombotic events in otherwise healthy individuals is less widely appreciated [3]. A comprehensive meta-analysis examining CMV-associated thrombosis identified nearly 100 reported cases in the literature, though the true incidence is likely underestimated due to under-recognition and underreporting [7]. A prospective cohort study demonstrated that acute CMV infection is associated with a significantly increased short-term risk of venous thromboembolism, with most events occurring within 30 days of acute infection [8]. Furthermore, a systematic review found that in immunocompetent patients with CMV-associated VTE, SVT, and splenic infarction occurred in approximately 50% of cases, highlighting the predilection for unusual thrombotic sites [9].

The pathophysiology underlying CMV-associated thrombosis is multifactorial. CMV has been shown to directly infect vascular endothelial cells, leading to endothelial dysfunction and loss of normal anticoagulant properties [5]. The virus induces expression of tissue factor and plasminogen activator inhibitor-1, creating a prothrombotic state [10]. Additionally, CMV infection triggers an inflammatory cascade with elevated levels of proinflammatory cytokines, including interleukin-6 and tumor necrosis factor-alpha, which further promote coagulation [5]. Some patients develop transient antiphospholipid antibodies during acute CMV infection, though these were absent in our patient [4].

Portal vein thrombosis in the absence of cirrhosis, malignancy, or inherited thrombophilia should prompt consideration of infectious etiologies, particularly CMV [6]. The combination of abdominal pain, fever, elevated liver enzymes, lymphocytic leukocytosis, and splenomegaly with splenic infarcts in a young patient strongly suggests CMV infection [2]. The three-week prodrome of flu-like symptoms in our patient likely represented the initial viremic phase of primary CMV infection, with subsequent development of thrombotic complications [3].

Management of CMV-associated thrombosis involves both anticoagulation and consideration of antiviral therapy. While anticoagulation is standard for the treatment of venous thrombosis, the role of antiviral therapy in immunocompetent hosts remains controversial [8]. Many experts recommend antiviral treatment with ganciclovir or valganciclovir for patients with severe disease manifestations, high viral loads, or evidence of end-organ involvement, as was the case with our patient, who had hepatitis, splenomegaly with infarction, and significantly elevated CMV PCR [9].

The duration of anticoagulation for CMV-associated thrombosis is not well-established. Most experts recommend at least three to six months of therapeutic anticoagulation, similar to the treatment of provoked VTE [6]. Some authorities suggest considering extended or indefinite anticoagulation for patients with thrombosis in unusual locations such as splanchnic veins [9]. The absence of inherited thrombophilia in our patient suggests the thrombosis was provoked by the acute CMV infection, supporting a finite duration of anticoagulation therapy.

This report emphasizes several important clinical pearls. First, CMV infection should be included in the differential diagnosis of fever, abdominal pain, and elevated liver enzymes, particularly when accompanied by lymphocytic leukocytosis [2]. Second, thrombotic complications can occur in immunocompetent patients with CMV infection, especially involving unusual sites such as the portal and mesenteric veins [7]. Third, prompt diagnosis through serologic testing and CMV PCR is essential [1]. Finally, combined treatment with anticoagulation and antiviral therapy should be considered in patients with severe manifestations or high viral loads [8,9].

## Conclusions

This report describes a case of superior mesenteric and portal vein thrombosis with splenic infarction caused by acute CMV infection in an immunocompetent young adult, emphasizing that CMV should be considered in patients presenting with unusual venous thrombosis, particularly in splanchnic vessels, even without traditional risk factors. Diagnosis requires integrating clinical features (fever, abdominal pain, elevated transaminases, atypical lymphocytosis, thrombocytopenia), serologic testing (CMV-specific IgM antibodies), CMV PCR quantification, and contrast-enhanced CT imaging to detect thrombosis and complications. Management combines therapeutic anticoagulation as the cornerstone with selective addition of antiviral therapy for severe manifestations, high viral loads, end-organ damage, or critical vessel involvement. The key clinical lesson is that the absence of traditional thrombophilic risk factors in young patients with atypical venous thrombosis, especially splanchnic vessels, should prompt evaluation for infectious causes like CMV, as early recognition and appropriate dual therapy with anticoagulation and antivirals can prevent serious complications and improve outcomes.
